# Physiological Psychology

**Published:** 1856-04-01

**Authors:** Robert Dunn


### Art V.—PHYSIOLOGICAL PSYCHOLOGY.

BY ROBERT DUNN, F.R.C.S. ENG.

It is no longer a subject of dispute, that the doctrines of mind rest essentially on the basis of our physiological composition—that they form a part of the physiology of man. For, however it may be attempted to separate intellectual and moral from animal and corporeal man, and however we may reason about our intellectual and moral nature apart from our bodily and animal constitution, it is never to be forgotten that they are united in this life, forming one and a composite system of mutual dependence and reciprocal action. From the first moment that *the primitive cell-germ* of an human organism comes into being, and is launched upon the ocean of time and space, it may literally be said, that the entire individual is present, that an organized entity exists, fitted for a human destiny; and that, from the same moment, *matter* and *mind*, *body* and *soul*, are never for an instant separated. Their union constitutes the essential mode of our present existence, and they are alike subject to the laws of development and growth; for the mind, like the body, passes through its phases of development. Not only is the framework and different organs of the human body evolved and perfected, one after the other, in accordance with all the subsequent wants of the future man; but, among the rest, and from the same primitive cell-germ, are gradually developed, the nervous apparatus and the encephalic ganglia, upon the vesicular matter of which the mind is dependent for the manifestation of all its activities. And thus we see, that in the primitive cell-germ of the human organism are potentially contained the *vital, nervous,* and *mental forces;* and, than the attempt to investigate and trace the genesis and gradual development of these forces, and their correlations with each other, what subject, to the psychological inquirer, can be more interesting or more important?*

The phenomena of the vital force are first displayed. For in the cell-germ, duly supplied with the nutrient pabulum, inherent are the powers of self-development and life under which the human fabric is evolved and built up. But after birth, to the organic processes, the animal functions and their allied appetites and instincts are superadded; and with these, sensations, as subjective conditions, are inseparably connected. Man then enters upon a new state of being and an individuality—an independent existence is established. For as soon as embryonic life is passed, the *nascent consciousness* becomes awakened,—roused into activity by stimuli from without, the senses coming into play from the moment of birth.

Now, consciousness is an *ultimate fact* in animal existence, beyond which we cannot go; it is the *distinguishing* attribute of animal life, the first of the phenomena of the *mental force,* and *self-consciousness* is the primary condition of intelligence: in a word, *it is mental existence.*

The great and fundamental mystery of life, indeed, consists in the relations of consciousness and of that dynamical agency which we designate *volition,* or the *will,* to the functions of the special senses, and those of the encephalic ganglia, which connect man as a sentient, percipient, and intelligent being, with his own bodily organization and with the world without. For, while it is no longer a matter of dispute that the encephalon is the material organ of the mind, where the ultimate molecular changes precede mental states, and from whence the mandates of the will issue, it has been well observed, by an acute metaphysician, that,—

"As to the nature of the relation which exists between the encephalon and the sentient and percipient mind, we never shall be able to understand more than is involved in the simple fact, that a certain

* The subject has engaged the attention of one of the ablest physiologists and most profound thinkers amongst us—I mean Dr. Carpenter. See his valuable paper, in the "Philosophical Transactions of the Royal Society," On the Mutual Relations of the Physical and Vital Forces; and his chapters On the Correlations of Physiology and Psychology, in the last and fifth edition of his "Human Physiology."

affection of the nervous system precedes immediately a certain affection of the mind. And that a peculiar state of the particles of the brain should be followed by a change of the state of the sentient mind is truly wonderful; but, if we consider it strictly, we shall find it by no means more wonderful than that the arrival of the moon at a certain point in the heavens should render the state of a body on the surface of the earth different from what it otherwise naturally would be. We believe, and, indeed, with as much confidence, that one event will uniformly have for its consequent another event, which we have observed to follow it, as we believe the simple fact that it has preceded it in the particular case observed. But the knowledge of the present sequence, as a mere fact to be remembered, and the expectation of similar future sequences, as the result of an *original law of our belief*, are precisely of the same kind, whether the sequence of changes be in the *mind* or in *matter*, singly or reciprocally in both."*

The essential nature of mind is a problem which belongs to the same category as the nature of life. We know nothing of life apart from organization; and we have no evidences of mind independent of a brain and nervous system. An organism is required for the display of vital phenomena, and an encephalon for the manifestations of mind. Life has accordingly been defined as "the collective expression for a series of phenomena which take place exclusively in bodies that are organized," and mind as "the functional power of the living brain."

But be it remembered, in affirming that sensation, perception, emotion, thought, and volition are functions of the nervous system, it is only maintained that the vesicular matter of the encephalic ganglia furnishes the material conditions, the substratum through which these mental phenomena are manifested, and that at the same time it is fully admitted the *essential phenomena of matter and mind* are so completely antagonistic, it is in vain that we attempt to establish any relationship of analogy or identity between them. But we have more satisfaction in the consideration of *mind*, in the light of *force*, and in the contemplation of the correlations of the forces of the physical, vital, nervous, and mental, for we see that the *nervous* and *mental* forces are constantly interchanged and interchangeable. We note the perpetually-recurring metamorphosis of *nerve-force* into *mind-force*, and of *mind-force* into *nerve-force*, and the important *physiological fact* that the nervous matter of the cerebrum is the material substratum through which the metamorphosis is effected. Nay, more, we have actual proof of *an increased disintegration* of the nervous tissue in the *redundant amount* of the *alkaline phosphates* in the urine, when the centre of intel-

* Dr. Brown's "Lectures on the Philosophy of the Human Mind." Lecture XIX.

lectual action has been over-taxed. And in all our voluntary movements and volitional acts we see the dynamical agency of *mind* producing *motion*, and that of the will, through the instrumentality of the *nerve-force*, acting upon the muscular system.

Dr. Carpenter has well observed :—

"We have evidence in what we know of the physiological conditions under which *mind* produces *motion*, that certain forms of the *vital force* constitute the connecting link between the two; and it is difficult to see that the dynamical agency which we term *will* is more removed from *nerve-force* on the one hand, than *nerve force* is removed from *motor force* on the other. Each, in giving origin to the next, is itself suspended, or ceases to exist *as such*, and each bears in its own intensity a precise relation to that of its antecedent and its consequent. But we have not only evidence of the excitement of *nerve force* by *mental agency;* the converse is equally true, *mental activity* being excited by *nerve-force.* For this is the case in every act in which our consciousness is excited through the instrumentality of the sensorium, whether its conditions be affected by impressions made upon the organs of sense, or by changes in the cerebrum itself, a certain condition of the nervous matter of the sensorium being (we have every reason to believe) the immediate antecedent of *all consciousness*, *whether sensational or ideational.* And thus we are led to perceive, that as the power of the will can develop nervous activity, and as nerve force can develop mental activity, there must be a *correlation* between these two modes of dynamical agency, which is not less intimate and complete than that which exists between the nerve-force on the one hand, and electricity and heat on the other. This idea of *correlation* of force will be found completely to harmonize with those phenomena which indicate the influence of physical conditions as the determination of mental states, whilst, on the other hand, it explains *that relation* between emotional excitement and bodily change which is manifested in the subsidence of the former, when it has expended itself in the production of the latter."*

Now, of consciousness as an ultimate fact in animal life—the first of the phenomena of the mind-force—we can best conceive in relation to time, as an incalculably rapid succession of acts or states, and as passing through a series of successive developments from the moment of birth. Purely sensational at first, it emerges gradually, *step* by *step*, from self-consciousness, through the perceptive and emotional to the higher phases of intellectual consciousness, until the mind reaches its dominant development in the perfect freedom of volition or the will.

But these progressive phases of mental development are dependent for their very existence upon the evolution and material

* "Human Physiology," pp. 553, 554. Fifth Edition.

condition of the vesicular matter of the encephalic ganglia through which they are manifested.

Comparative psychology, the study and strict interpretation of "the living experiments (to use the happy and expressive language of Cuvier) which nature has presented to us in an ascending series, in the varying forms of animal existence," from the lowest up to man, not only establishes the fact that sensation, perception, emotion, and intellectual action, are distinct states of consciousness, successively developed, but that these states are manifested through different portions or nervous centres of the encephalon, and that the human mind in its progress to maturity passes through these successive phases of development.

Man is at birth the mere creature of sensation and instinct, so that sensational consciousness and consensual and instinctive actions constitute the earliest stage of his psychological existence. The senses come into play from the moment of birth, and they soon acquire the utmost perfection of which they are capable, but their intentions are strictly consensual.

All our actions are automatic, reflex, consensual, and instinctive, until the perceptive consciousness has been developed. But with the perceptive consciousness we have its associate memory, and the genesis and development of the will. We attain to the free exercise of volitional power, and to the performance of purely voluntary actions. With the perceptive consciousness emotional sensibility is indissolubly connected; for we see the expression of joyous emotion in the infant's laughing eye, as the perceptive consciousness begins to dawn, and as the powers of recognition and volition are awakened; and though long before we can reason or reflect we manifest the emotional and social instincts, no one can take upon himself to say at what precise moment the infant eye ceases to convey a mere nervous impulse, and when it awakens in the mind the first glimpses of the sublime and beautiful.

The ratiocinative and reflective consciousness are the last developed and the latest to reach maturity. As sensation is the earliest and lowest, so is ratiocination the latest and the highest of our mental attributes.

*Sensational Consciousness.*—Sensation is the link in the chain of being between the vital and mental forces, connecting indissolubly together the conscious and the unconscious processes. As a complex act it lies partly within and partly without the consciousness, and as soon as embryonic life is passed, it traverses the line which separates the physical and vital from the nervous and mental processes, enters the light of consciousness, and thus becomes a fact, psychological as well as physiological.

As a subjective condition, sensation is identical with simple consciousness, and the two great and distinguishing functions, typical of animal life, namely, sensation and locomotion, are seated in the sensory and motory ganglia of the nervous system.

Now, sensori-motor, consensual, and instinctive feelings and actions formularize the sensational consciousness; and in its simplest but essential type, the nervous apparatus of the sensori-motor, or sensational consciousness consists of a series of nervous centres or ganglia, and of internuncial conductors, or of commissures and nerves. The vesicular matter of the nervous centres or ganglia is the source of the nervous force, where impressions are received and impulses are generated; between these centres the commissures are the media of communication, and to and from them the nerves are internuncial conductors or cords.

In the invertebrate subkingdom is best seen the simplest form or apparatus of the sensational consciousness, namely, distinct ganglia, with commissural cords and nerves, administering to the functions of automatic life and to the operations of instinct. But in the nervous system of the invertebrata, we have the homologues of the sensational consciousness of the vertebrate series, for the sensory ganglia are the seat of the sensational consciousness of whatever kind, and the cranio-spinal axis the source of all the movements of the body, the two great centres of sensation and motion being brought into harmonious and associated action through the medium of the cerebro-spinal axis. In man, and throughout the whole of the vertebrate subkingdom, the sentient and sensori-motor apparatus, the system of automatic life, and instinctive action subservient to sensations, and to those consensual and instinctive actions which are indissolubly linked-on with sensations, consist of the spinal axis and nerves, the medulla oblongata, and the chain of sensory ganglia, including those of the special senses at its summit. For if we follow up the cranial prolongation of the spinal cord, the medulla oblongata, into the fibrous strands of which we see imbedded the *respiratory*, *auditory*, and *gustatory ganglia*, and carefully trace out its ramifying branches, we find it sending off distinct fasciculi of fibres to the ganglionic centres at its summit, to the cerebellum, the corpora quadrigemina, the thalami optici, the corpora striata, and to the peduncles of the olfactory ganglia. And thus we see, to the *sole exclusion of the cerebrum*, whose connexions are *strictly commissural*, that the whole series of the ganglia of the cerebro-spinal system, including those of the special senses, are in direct fibrous connexion with the cranio-spinal axis, and form with it, as an aggregate or whole, the *sensorium commune*, or *great circle of sensational consciousness and of consensual and instinctive action.*

Now, the sensori-motor, consensual, and instinctive phenomena, which formularize the sensational consciousness, though the lowest in the psychical scale, are independent of, and ought not to be confounded with, intelligent and volitional actions. For while we recognise in the nervous apparatus, through which these phenomena are manifested, the homologues of the sensational consciousness of the vertebrata, and even of man himself, we find that the motor centres of the articulata, and the sensory of the mollusca, are alike destitute of those crowning and special ganglia, *the cerebral hemispheres*, which are the seat of the perceptive consciousness, of intellectual action, and volitional power. It is admitted that there is no point in physiology more clearly made out than that the cerebrum, or great hemispherical ganglia, is the centre of intellectual action and volitional power, the seat of the understanding and the will.

But in myriads of animals, indeed in the whole of the invertebrate subkingdom, with the exception of the highest mollusca, the cuttlefish, *no cerebrum exists;* and, even in the lower vertebrata, the olfactory, optic, and auditory ganglia have no direct connexion with it, so that the totality of their life is made up of sensational consciousness, and of reflex, consensual, and instinctive actions. And such, too, is the primitive condition of man in the first stage of his existence, for at birth all his acts are reflex, consensual, and instinctive, and generally among the first roused into activity by the effects of the external stimuli of his environment, is that of crying very lustily; and next to this follows the untaught motions of the lips in the act of sucking, to satisfy an internal want and instinctive feeling. The instincts, the untaught activities and capacities of our animal nature are *innate.* As internal subjective feelings, they arise in obedience to certain laws of our nature, or are brought into play in direct respondence to stimuli acting upon the sensational consciousness from without. The infant mind responds solely at first to impressions from *without*, or from instinctive feelings from *within.* The sudden light, indeed, may dazzle, and a loud noise may startle; but until the perceptive consciousness has been awakened, the mind is in a state of isolation,—it takes no cognizance of an *outward world.* To it the inward world is everything, and the outward world is nothing. Its sensations are all subjective, and its actions reflex, consensual, and instinctive. But even in adult life the functions of the cerebrum may become suspended, and man reduced to his primitive condition of mere sensational and instinctive being. When the functions of the cerebrum are thus benumbed and paralysed, and when it is no longer capable of receiving and acting upon sensorial impressions, it is then that the sensory ganglia, as an independent centre of action, becomes so strikingly manifest. An interesting and instructive instance of this kind was for some months under my observation about ten years ago. But having published the narrative, with a commentary on some of the most important of its psychological bearings, I need here merely observe, it was the case of a young woman, in whom the intellectual faculties were quite suspended, and whose only open avenues to the sensational consciousness were those of *sight* and *touch*, for she could neither hear nor speak, taste nor smell.*

Among the functions of the sensational consciousness, common sensibility or feeling, and the capability of experiencing pleasure and pain from mere tactile impressions, are primordial, the most universal in nature, and the most essential to human existence. Some, indeed, have maintained that all the other special senses are but modifications of that of touch. This notion of Democritus, of which the fallacy is obvious, we can readily conceive had its origin in the observed fact of the *necessity of contact* in the operations of all the senses, between the physical impulse and the external organ of sense. Thus in sight, where the eye is the organ, and light the medium, the rays must impinge upon the retina; and in hearing, the vibrations of the air must strike upon the tympanum. So, too, in taste and smell, the sapid and odoriferous particles must be brought into actual contact with the papillæ of the tongue, and the pituitary membrane of the nose. But all this merely points to a community of action in their mode of operation, whilst the fallacy consists in overlooking the all-important fact, that each of the sensory ganglia of the external senses is the seat of a *special endowment*, and that each conveys to the sensational consciousness a different kind of intelligence. Thus, when electricity is brought to bear upon the eye, it excites a consciousness of light, upon the ear of sound, upon the nose of smell, upon the fingers of a prickly feel, and upon the tongue of an acid or alkaline taste. These functions are not interchangeable.

"The eye cannot detect the noxious atoms arising from a putrid animal; the ear is unaffected with the contents of its own ministering fluid, however heavily laden with scents; the hand cannot finger the fragrance of the rose, nor the tongue taste one of the hundred perfumes which may be served up in a parterre of flowers."†

We cannot see with our tactile organs, hear with our visual, nor smell with our auditory.

* "Physiological Psychology. A Case of suspension of the mental faculties, of the powers of speech, and special senses, with the exception of sight and touch, continuing for many months; with a Commentary on some of the more important of its bearings, upon the Philosophy of the Human Mind and the Physiological Psychology of Man." By Robert Dunn, F.R.C.S. Eng. London: T. Richards, 37, Great Queen-street. 1855.

† "British Quarterly Review." April, 1855.

*Special Senses.*—The external senses have been emphatically styled the "Alphabet of Intelligence." They are the inlets to the materials of knowledge, and constitute, with their allied, consensual feelings, appetites, and instincts, the inferior region of the true or conscious mind. They occupy a prominent, not to say predominant, part of the mental life, of the great mass of the inferior animals, and a very considerable portion of the far more complicated thread of human existence. Each of the sensory ganglia of the special senses, in the encephalon, conveys to the sensational consciousness a different kind of intelligence, and they are obviously the seat of the simple feelings of pleasure and pain, inseparably connected with the exercise of their functional endowments, as well as the centres and source of those motor impulses, which once evoked, react upon the muscular system, independently of volition or thought. But the intuitions of the senses are strictly consensual, and confined to the sensible phenomena of matter, without conveying to us any knowledge whatever of the bodily substance with which they may be connected. Thus we see light, we hear sound, we smell odour, we taste sapor, and we feel pain, heat, or cold. To these intuitions Oken has given characteristic designations. He calls touch or feeling the *earth sense;* sight, the *light sense;* hearing, the *motion sense;* smell, the *air sense;* and taste, the *water sense.*

Mr. Wedgwood has well observed :—

"It is hardly necessary to premise that we have no knowledge of *body* by any of the five senses. What I immediately perceive by sense is the *sensible phenomena* itself, and not the bodily substance with which it may be locally connected, either as the proximate cause of the sensation, or as the organ by or in which it is felt. When I suffer toothache, or when a pin is run into me unawares, the thing of which I have actual apprehension is the pain I suffer, not the bodily substance of the pin and the tooth. When a gun goes off before my window, what I hear or perceive by the ear is neither the bodily gun nor the vibrations of the air, by which the material action is conveyed to my ear, but the sound itself. When I gaze upon the stars, the visible image before my eyes affords a subject for contemplation, apart from all speculation as to the bodily nature of the object seen. Thus the exercise of the senses displays to us five elementary *modes of being,* logically unconnected with the bodily substance. Five kinds of being upon which we may think, independent of all intellectual reference to bodily support."*

The special senses have been classified and grouped in the order of their importance into the superior or *psychical,* and the inferior or *animal,* the former comprehending feeling, seeing, and hearing, and the latter taste and smell. This arrangement

* "Cambridge Philosophical Transactions." Tract by H. Wedgwood, Esq., quoted by Mr. Morell, "Elements of Psychology."

is in perfect accordance with the varying character of the commissural relations of their sensory ganglia in the encephalon, and is strikingly exemplified in that of feeling or touch.

*Touch.*—Of all the special senses, touch is the most important, for it is the most essential to human existence. It is the most universal in its application, and forms the starting-point to all the rest, combining to a certain extent all their functions, and enabling us gradually to replace the loss of the other senses, by manifold comparisons, but being itself never replaced by any combination of them. Its mode of action is best illustrated by the simple notion of *resistance*, and it is through its agency that we acquire distinct conceptions of the physical qualities of bodies, such as their hardness, softness, roughness, smoothness, &c.

As the peripheral extremities of all the different spinal nerves, diffused and ramifying upon the entire superficies of the body, administer to the sense of touch, and as these impressions are transmitted from the posterior segmental ganglia of the spinal cord upwards along the sensory tracts to the thalami optici, it has been legitimately inferred that these bodies are the encephalic ganglia of tactile and common sensation. Still, as the sense of touch is both *subjective* and *objective* in its bearings—at one time the source of physical pleasure, and at another the awakener of intellectual ideas,—the subjective phenomena have been separated from the objective, or tactile sense, and designated, *par excellence*, *feeling*, or *common sensation;* and while this has been treated in the thalami optici as its encephalic centre, the tactile sense has been referred to the corpora dentata* of the cerebellum, and which, it must be admitted, are in direct commissural connexion with the posterior segmental ganglia of the cord along which the tactile impressions are transmitted.

Pathological researches have produced the conviction in my own mind that the corpora dentata are the seat of the *muscular sense*, and the thalami optici, that of *common sensation* and *tactile feeling*. We cannot deny to the little lancelott (*the amphioxus*) which may be viewed in the light of a connecting

* By Dr. Noble, of Manchester. Vide his "Elements of Psychological Medicine" As bearing upon this point, I would refer to a "Case of Tubercles in the Brain, with Remarks, Physiological and Psychological, on the functions of the Nervous Centres involved in the Disease," which I published in the *Association Medical Journal* (pp. 712–16). 1854.

At the autopsy, there was found in the lateral lobe of the cerebellum on the left side, a mass of *tubercular deposit* a little to the outer side of the median line, in a state of softened degeneration, and during life the following diagnostic phenomena were noticed in the child:—"There was an imperfect paralysis of the right side, both of the arm and leg, but still they responded to the mandates of the will: she could move her arm about, and could grasp anything firmly enough in her right hand, *when her eyes and attention were directed to it;* but, *if they were diverted to something else, and the volitional power withdrawn, she would let the object which she had been holding fall from her hand, and without being conscious of the fact.*"

link between the vertebrate and invertebrate series of animals, the possession of *common sensation* or *tactile feeling;* and yet we know that, in its case, no cerebellum exists, and, in consequence, that the corpora dentata are wanting. But the thalami optici are not simply the great encephalic centres of common sensation, where the sensory strands of the medulla oblongata terminate. They are, in reality, the common centre and point of union of the sensory nerves. Implanted upon the sensory tracts of the crura cerebri and medulla oblongata, they are in direct fibrous commissural connexion with the respiratory, gustatory, and auditory ganglia, and with the optic nerves, by a direct passage of a portion of their roots, and with the peduncles of the olfactory nerves, through the medium of the fornix; so that a connecting nervous thread ramifies throughout the entire circle of special sensation, and the thalami optici form a *common foci* and *point of union* to all the nerves of special sense. And this harmonizes well with the universality of the feeling, or common sensibility, which pervades the entire system, and which is associated with all the voluntary movements of the body, and the exercise of the functions of all the other special organs of sense.

"Without some point of union, some fixed reality, running like a continuous thread through all the phenomena of the special senses," it has been well observed by Mr. Morell, that, "our whole sensational life would be a succession of mere impressions, each point of existence being distinct from the other, and each removed sensation like a momentary life and death of the whole individual. In this chaos of impressions, accordingly, and around a middle and uniting point, they all tend to cluster; order begins to ensue; a connexion between the phenomena of the different senses manifests itself, and the shadow of a continuous life, of which these impressions are but the passing phases, is projected from out of the dark confusion.

"This shadow is the first rise of *self-consciousness*—the middle point of our phenomenal existence—the unity around which all our sensations, from the earliest period, are gradually marshalled. Accordingly, the primary form of self-consciousness is the *unity of sense.*"*

And thus, as the encephalic ganglia of common sensation, and the centre and point of union of the nerves of special sense, we see what an important part the thalami optici play in the great circle of sensational consciousness and instinctive action. But their office does not end here. When we come to the consideration of the perceptive consciousness, from their intimate relations with the cerebrum and corpora striata, the centres of intellectual action and voluntary motion, we shall find what an important office the thalami optici sustain in operations which rank high in the psychical scale.

* Morell's "Elements of Psychology."

*Sight and Hearing.*—Next to touch or feeling, sight and hearing are the most important of the senses. They have been viewed in contrast. Intellectually, *sight* is *knowledge*, and *hearing, attention.* To *see* is to *know*, and to *hear* is to *listen.* Sight is the clearest and hearing the deepest of the senses. The visual impressions on the retina pass at once to the perceptive consciousness, while the pathos of the orator appeals directly to the deepest feelings and emotions of the soul. Hearing is feeling, and tone moves us. An external object shows its outward surface; but it is the tone or sound which it sends forth that betrays what exists within. It is not the form or colour of an object which tells what it is, but its sound.

*Sight.*—Sight is the highest, most refined, and objective of all the senses; for sight is knowledge. I need not dwell upon the importance of the sense; but, at first, it is nothing more than an overpowering sensation of light, no object being individually distinguished.

The corpora quadrigemina are the encephalic centres of vision, though some physiologists have restricted the function to the corpora geniculata; and it must be admitted that, like the thalami optici, the corp ora quadrigemina have a higher and wider sphere of action; for, like them, they are associated with our emotional states, and they are not the mere ganglia of sight; their commissural relations in the encephalon are commensurate with the importance of their functions, and their direct connexion with the seat of the muscular sense,—the corpora dentata in the cerebellum,—is just what *à priori* reasoning would lead us to expect, seeing how invariably in health all our voluntary movements are under the direction and guidance of *sight* as well as *feeling.*

*Hearing.*—The sensory ganglia of the auditory nerves are embedded in the posterior pyramids, in the sensory tracts of the medulla oblongata, and their commissural relations well accord with the psychical and emotional character of the sense of hearing. It is more subjective than that of sight,—for hearing is feeling, and it administers more largely to our sensuous feelings. We hear succession—continuous sound being the result of a succession of impulses, communicated from without to the auditory nerves. It is a refined kind of touch. Objectively considered, hearing is motion; intellectually, it is *attention;* for to *hear* is to *listen.* It is one of the most important inlets to knowledge, and is associated with our instinctive, emotional, and intellectual states. The sensations of sound, when wrought into music, are the sources of infinite pleasure; and who can estimate the marvellous influence of articulate speech!—the magic of the human voice!

"The world of sounds is scarcely less important than the world of sights. All the rich varieties of tone, all the diversified notes of nature; from the whisper of the wind to the crash of the thunderbolt; from the massive harmonies of Handel, to the gentle wail of the parting spirit as it sings the flesh to sleep!"*

*Taste and Smell.*—Taste and smell are the animal senses, and are intimately connected with the organic sensations of the alimentary canal. They administer to the most important purposes of animal life, but are more subservient to our physical comforts and welfare than to our intellectual development.

Taste is the chemical sense, and the gustatory ganglia, through which we acquire a knowledge of the sapid qualities of substances, are embedded in the sensory strands of the medulla oblongata with the nuclei of the glosso-pharyngeal nerves and a portion of sensory roots of the fifth. Of all the senses, taste is most allied to that of touch, or common sensation. It has no special nerve of its own, like smell, or sight, or hearing. But it is proved beyond dispute, that the *gustative impressions*, which excite nausea and vomiting, are conveyed to the medulla oblongata exclusively through the glosso-pharyngeal nerves; and as nausea and other tastes do become idealized, the sensory impressions must pass up to the thalami optici, and through those channels reach the perceptive consciousness.

Taste penetrates to the chemical constitution of bodies, and it has for its object the selection of food, and the excitation of the flow of the saliva. It is the steward of the stomach, and smell is the guardian of the lungs. They are co-operators.

"The toil of eating is a pleasure; a sense is stationed at the gateway of the alimentary canal, and endowed with the power of enjoying the substances required by the frame for its support. When a sapid substance comes in contact with the tongue, its papillæ rise up like a little army, as if to examine the intruder. This Inspector-General of Taste divides them into three classes, the *insipid*, *unpalatable*, and *positively agreeable*. It tells us how the external world *tastes*, and right skilfully does it do so. A beautiful accord subsists between the tongue and the stomach. The warden of the lodge knows what visitors will visit his colleague in the hall of digestion. Rightly used, the sense of taste is a gift of the most benevolent description."†

*Smell* is the air sense. Like a sanitary guardian at the portal of the lungs, it tests the purity of the air we breathe, and is closely allied to taste.

"As sentinel at the gateway, it reports to the mind when it finds any suspicious perfumes are abroad. The nose is the official inspector of nuisances. It strains the air for the lungs, and it tests the poison which may be suspended in that essential fluid."‡

* "British Quarterly Review," April, 1854. † Ibid. ‡ Ibid.

The instinct of *self-preservation* is the most universal instinct in nature, and the very first that is roused into action. To it all the special senses are subservient, but first and foremost, those of smell and taste. Smell is its first and guiding sense, for it is the sense of smell which attracts and guides the human infant to the mammary gland of its mother to satisfy an *internal want* or *craving*.

I have alluded to the commissural connexions of the peduncles of the olfactory nerves with the thalami optici, the *great centres of sensorial feeling*, the foci and point of union of all the nerves of special sense, but they are also directly connected with those primitive basilar convolutions of the cerebrum which surround the *fissura Sylvii*, and which are coeval in point of existence with the fissure itself.

The impressions both of taste and smell become *idealized* and registered. We all know by experience how a savoury odour will cause the mouth to water, and how a noxious and disgusting effluvia will induce nausea and a sickening feel; but is it not equally true that the very thought of them, the mere recollection of the *idealized sensations*, will produce the same effects?

But besides the impressions from the special senses, and the simple feelings of pleasure and pain associated with the exercise of the functional endowments of their sensory ganglia, there are various classes of sensations and subjective feelings appertaining to our bodily states which are brought under the cognizance of the sensational consciousness;—such are the sensations of organic life, and of the appetites and instincts. With the animal processes sensations are inseparably connected, and with sensations their allied appetites and instincts. But sensations are the primary phenomena, and form the starting-point to the other two; for it is obviously manifest that an appetite or instinct must be preceded by or attended with a sensation. Sensations are either pleasurable or painful, but pain is the exception, and the indirect, and not the direct effect of the actions of life.

There is the pleasurable consciousness which constitutes the feeling of health, but there is also a feeling of sickness, of lassitude as well as of vigour, and a great variety of painful subjective feelings, arising from particular states of the muscles, as shuddering, twinging, spasms, cramp, &c., and which are conveyed by afferent nerve fibres to the sensorium, and thence to the sensational consciousness, awakening a consciousness of our bodily states.

There is a general feeling of *well-being*, and one of *malaise*, in common language known as "the state of the spirits;" and this state of *self-feeling*, or *canæsthesis*, is of a varying character, and greatly influenced by the bodily temperament. Some there are—

"So keenly susceptible of both conditions, that they pass their whole lives in an alternation between *cheerfulness* and *depression*, the former state being favoured by freedom from anxiety, by the healthy activity of all the organic functions, by a bright sun, and a dry bracing atmosphere, whilst the latter is immediately induced by mental disquietude, by a slight disorder of digestion or excretion, or by a dull, oppressive day. In such individuals, favourable conditions may even exalt the canæsthesis into *exhilaration* or *absolute joy*, whilst the combined influence of opposite conditions may produce *gloom*, which may be exaggerated almost to *despair*. The condition of 'the spirits' most to be desired is that of *tranquil comfort*, for this is far more favourable than the alternation of extremes to healthful activity, and to sustained energy both of body and mind."*

(*To be continued.*)

---

### Art. VI.—ON MORAL AND CRIMINAL EPIDEMICS.

* Dr. Carpenter's "Human Physiology."

Sir Henry Holland has published some admirable papers—On the Effects of Mental Attention on Bodily Organs, in his "Medical Notes and Reflections," and in his chapters on Mental Philosophy, well worthy of the attention of the medical philosopher and observant practitioner.

